# Vector-Borne Bacteria Detected in Ticks, Mites and Flies Parasitizing Bats in the State of Rondônia, Brazilian Amazon

**DOI:** 10.3390/pathogens14040338

**Published:** 2025-03-31

**Authors:** Leormando Fortunato Dornelas Júnior, Irineu Norberto Cunha, Felipe Rodrigues Jorge, Gustavo Graciolli, Ricardo Bassini-Silva, Fernando de Castro Jacinavicius, Maria Carolina A. Serpa, Marcelo Bahia Labruna, Felipe Arley Costa Pessoa, Luís Marcelo Aranha Camargo

**Affiliations:** 1 Leônidas and Maria Deane Institute, Manaus 69057-070, AM, Brazil; felipe.pessoa@fiocruz.br; 2 Institute of Biomedical Sciences, University of São Paulo, Monte Negro 76888-000, RO, Brazil; 3 Instituto Butantan, São Paulo 05503-900, SP, Brazil; irineu.cunha81@gmail.com; 4 Alto Tietê Ambiental, Mogi das Cruzes 08727-020, SP, Brazil; 5 Faculdade de Medicina Veterinária e Zootecnia, University of São Paulo, São Paulo 05508-270, SP, Brazil; felipecatundavet@gmail.com (F.R.J.); lilabiovet@gmail.com (M.C.A.S.); labruna@usp.br (M.B.L.); 6 Federal University of Mato Grosso do Sul, Campo Grande 79070-900, MS, Brazil; gustavo.graciolli@ufms.br; 7 Zoological Collections Laboratory, Butantan Institute, São Paulo 05585-000, SP, Brazil; ricardo.bassini@gmail.com; 8 Department of Animal Biology, Institute of Biology, University of Campinas (UNICAMP), Campinas 13083-970, SP, Brazil; fcjacinavicius@gmail.com; 9 Research Center of Rondônia (CEPEM), Porto Velho 76812-329, RO, Brazil; 10 National Institute of Epidemiology in the Western Amazon—INCT-EpiAmo, Porto Velho 76812-245, RO, Brazil

**Keywords:** Amazon, chiroptera, ectoparasites, ticks, mites, flies, vector-borne bacteria

## Abstract

Bats (Chiroptera) are among the most diverse and geographically dispersed mammals. They are of great importance to the ecosystem, as pollinators, seed dispersers and pest controllers, in addition to being hosts to several parasitic arthropods, including ticks, mites, lice, fleas and flies. Their diet includes the tissue and blood or other body fluids of bats. Bats are reservoirs of several disease-causing agents, many of them pathogenic to humans, such as bacteria, as well as protozoa, viruses and fungi. This study was conducted in Monte Negro, Rondônia, Brazil and the occurrence of parasitic arthropods in bats was evaluated, as well as a screening of bacteria that these ectoparasites can carry. Through a total of 69 nocturnal captures, 217 chiropterans were sampled, representing 23 species and six families. A total of 592 specimens of parasitic arthropods (ticks, mites and flies) were collected from these bats (9% dipterans, 59% ticks and 32% mites). *Bartonella* spp. were found in two species of bat flies (*Trichobius joblingi* and *Strebla mirabilis*) in peri-urban and forest areas with an infection rate of 62% and 38%, respectively. We report for the first time in Rondônia the argasid tick *Ornithodoros hasei* and its infection by a spotted fever group bacterium ‘*Candidatus* Rickettsia wissemanii’ in a peri-urban area.

## 1. Background

Among living mammals, Chiroptera is one of the most diverse orders, representing about 20% of all mammals in the world [1]. According to the most recent estimate, this order comprises 20 families and more than 1400 species [1,2,3]. Despite the recurring disturbances caused by human actions in nature [4], factors such as the development and popularization of new sampling methods, combined with the improvement of systematic reviews and the description of new species, have contributed to an exponential increase in the richness of known bats in Brazil. This keeps the country among the three with the greatest diversity of bats in the world [5], surpassed only by Indonesia with 230 species [6] and Colombia with 201 species [7]. In Brazil, nine families, 64 genera and 182 species are known, of which 150 species are found in the Amazon region of Brazil and 86 species with 16 genera in the state of Rondônia [8,9,10].

Bats have been reported as hosts of several arthropod ectoparasites, such as ticks (Argasidae and Ixodidae) [11,12], mites (Mesostigmata [13], Sarcoptiformes [14] and Trombidiformes [15]), lice (Phthiraptera) [16], fleas (Siphonaptera) [17] and flies (Diptera) [18]. In the Amazon, arthropods of the class Arachnida, three families, five genera and 60 species have been reported in bats and, in the state of Rondônia, three families, four genera and 16 species have been reported to date [12,15,19]. Regarding the class Insecta, 43 species in 16 genera of the family Streblidae and four species in two genera of the family Nycteribiidae have been reported [20,21].

The diet of these ectoparasites includes the blood or other body fluids and tissues of bats [13]. The presence of ectoparasites can cause irritation and stress to bats, which can affect their health and behavior [22]. However, the prevalence and diversity of these ectoparasites vary among bat species and their habitats [18]. Chiropterans and their ectoparasites are reservoirs for etiological agents of several diseases, and many of them are pathogenic to humans, such as bacteria of the genera *Rickettsia* [23], *Coxiella* [24], *Borrelia* [25], and *Bartonella* [26], as well as protozoa [27], viruses [28], and fungi [29].

According to Hayman [30] and Subudhi et al. [31], in recent years, interest in bat research has increased due to the occurrence of what is known as the spillover phenomenon, that is, the transmission of a pathogen from its natural reservoir or host species to a new host species, thus increasing the possibility of disease spread to humans and other mammals. In addition to bats, rats can transmit pathogenic bacteria to humans, such as *Leptospira* spp., *Yersinia pestis*, *Streptobacillus moniliformis*, *Spirillum minus*, *Arenaviridae group*, and the *Bunyaviridae group*.

Despite several studies in recent years, the study of ectoparasites in bats has been unevenly focused within a large geographic area of Brazil. The study by Graciolli and Bernard [18] formally recommended the exploration of areas such as south-central Brazil to carry out new inventories in the Amazon. Thus, the present study aims to record the pathogenic bacteria carried by the ectoparasitic fauna of bats in the south-central area of Rondônia in the western Brazilian Amazon.

## 2. Materials and Methods

### 2.1. Study Site

**Figure 1:**
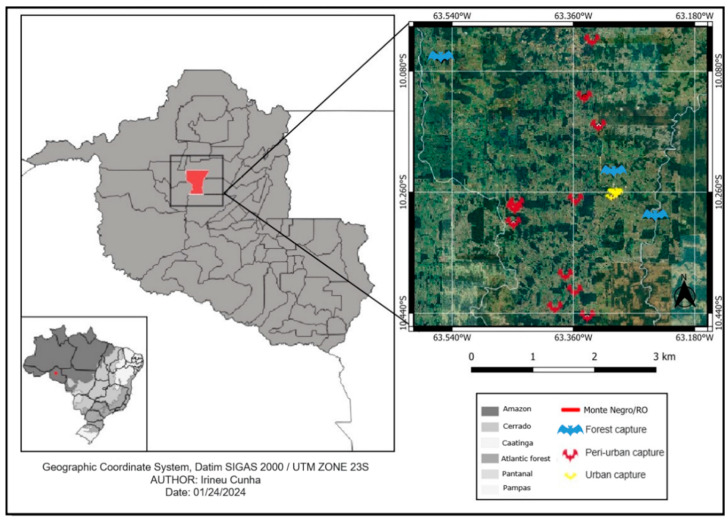
Sites where bats were captured in forest, peri-urban and urban areas in the municipality of Monte Negro, state of Rondônia, the western Brazilian Amazon.

This study was carried out between November 2020 and November 2022 in Monte Negro, a central region in the state of Rondônia, 250 km southwest of the capital Porto Velho. It is located at a latitude of 10°17′40″ S and longitude of 63°19′31″ W, at an altitude of 123 m, and occupies a territorial area of 1931.378 km^2^ with an estimated population of 11,548 inhabitants, according to data from the 2022 IBGE census (Figure 1). The region has mixed soils, equatorial vegetation and a hot and humid climate, with a high annual rainfall of 1440 mm (November to April) and 557 mm in the dry season (May to October). Average temperatures range from 25 to 29 °C, with relative humidity between 70 and 80% throughout the year [32]. The sites of bat capture were the forest (dense vegetation and low human density), a peri-urban area (characterized by having small sites and patches of forests with dense vegetation and low human density), and an urban area (diameter of 4 km^2^ per area, no forest, high human density and 12% empty buildings).

### 2.2. Bat Capture and Sample Collection

To capture the bats, we used ten mist nets measuring five meters long and two meters wide, suspended at a height of 1.5 m from the ground. Additionally, we conducted active research in artificial shelters in urban areas, resulting in the capture of 18% of the bats in the roof spaces of homes.

The mist nets were strategically positioned close to fruit trees and animal breeding areas, previously analyzed in terms of the location and behavior of bats in the environment. As necessary, these nets were occasionally relocated to meet the specific demands of the situation. To prevent escape or predation of captured specimens, the nets were inspected at 15-min intervals.

This procedure was carried out with extreme caution, aiming to avoid any damage or injury to the animals. After carrying out the procedures (biometric data collection processes, external morphology analysis and photographing), the bats were promptly sent to the fields to be released. Taxonomic identification of the bats followed specialized scientific literature [33,34,35]. The nomenclature of the species and the taxonomic arrangement followed Abreu Jr et al. [9].

Captures took place from 6:00 pm to 11:00 pm, over three consecutive nights, each month, during the waning moon (as suggested by the Butantan Institute protocol), for 25 months, totaling 345 h. According to Straube and Bianconi (2002), the capture effort, measured as the total area (m^2^) of the nets multiplied by the exposure time (hours), was 157,500 m^2^/h [35].

The bats were initially kept in individual cloth bags and subsequently handled and thoroughly examined for the presence of ectoparasites. These were removed using tweezers and placed in DNA-free microtubes containing 100% ethanol. Each microtube housed the ectoparasites collected from a single bat and was identified by a tracing paper label containing the animal’s code written in graphite pencil and inserted inside the tube. The molecular analysis of the bacteria in the ectoparasites studied is described below. No blood was taken from the bats.

### 2.3. Ticks, Mites and Flies

The ticks collected from the bats were morphologically identified at the species level following Jones and Clifford [36] and Labruna et al. [37]. In addition, 25 larval ticks were identified at the species level using molecular analysis. For this purpose, larvae were individually submitted to DNA extraction using the guanidine isothiocyanate phenol technique [38] and tested via a polymerase chain reaction (PCR) assay targeting a ≈ 460-bp fragment of the ticks’ 16S rRNA mitochondrial gene, as described by Mangold et al. [39]. The PCR products were purified and sequenced with a Big Dye Terminator Cycle Sequencing kit (Applied Biosystems, Foster City, CA, USA) in an automatic sequencer (ABI 3500 Genetic Analyzer, Applied Biosystems) according to the manufacturer’s protocol. The generated sequences were submitted for BLAST analysis (www.ncbi.nlm.nih.gov/blast, accessed on 3 March 2024) to infer the closest identities to the tick DNA sequences available in GenBank.

The mites were morphologically identified to species level following the keys available in Rudnick (1960) [40], Machado-Allison (1965) [41,42] and Herrin and Tipton (1975) [43]. All the other mites were under identification at the time of writing and will be identified to species level in future studies.

The bat flies from the families Streblidae and Nycteribiidae that were collected were identified using the key of Carvalho et al. [44], and were identified up to species level using the keys proposed by Guerrero [19].

### 2.4. Molecular Detection of Vector-Borne Bacteria

A sample of 87 ticks was selected for testing the molecular detection of bacteria of the genera *Rickettsia*, *Borrelia* and *Coxiella*. This sample included the 25 individual larvae mentioned above, plus 62 larvae that were processed individually (8 larvae) or in pools, each containing 2 to 3 individuals (a total of 19 pools containing 54 larvae), using the same DNA extraction protocol mentioned above. This procedure resulted in a total of 52 extracted DNA samples, which were initially tested using a TaqMan real-time PCR assay targeting the rickettsial citrate synthase gene (*gltA*), as described elsewhere [45,46]. Samples that were positive according to the real-time PCR (cycle threshold ≤35) were tested via a conventional PCR that targeted a 401-bp fragment of the *Rickettsia gltA* gene [45]. In each set of reactions, negative control tubes containing water and a positive control tube containing *Rickettsia vini* DNA were included. The PCR products were DNA-sequenced and submitted for BLAST analyses as described above.

For the detection of *Borrelia* DNA, all 52 tick samples were tested via a TaqMan real-time PCR assay that targeted the 16S rRNA gene of bacteria of the genus *Borrelia*, as described elsewhere [47]. Negative control tubes containing water and a positive control tube containing *Borrelia anserina* DNA were included. For the detection of *Coxiella* DNA, the 52 tick samples were tested via a conventional PCR that targeted a 687-bp fragment of the transposase elements gene (*IS1111*) of organisms of the genus *Coxiella*, as described elsewhere [48]. Negative control tubes containing water and a positive control tube containing *C. burnetii* DNA were included.

For the molecular analysis, DNA extraction of the mites was performed using the DNeasy Blood & Tissue kit (Qiagen, Hilden, Germany), following the manufacturer’s instructions. Each mite DNA sample was subjected to a conventional PCR targeting a fragment of ~800 bp of the 18S rRNA gene (endogenous control) [49] to verify the success of the DNA extraction procedure. Negative (Milli-Q water free of DNA) and positive (pool of dust mites) controls were included in each reaction.

One hundred and fifteen mites were selected for the detection of organisms of the genera *Bartonella*, *Rickettsia*, *Borrelia* and *Coxiella*. The protocols for the last three bacterial genera were the again used for the ticks. For *Bartonella*, we used a conventional PCR targeting a fragment of the *nuoG* gene of *Bartonella* spp. according to Colborn et al. [50].

All PCR products with concentrations over 20 ng/µL were selected and purified with ExoSap-IT (GE Healthcare Pittsburgh, PA). Sanger sequencing was performed at the Centro de Pesquisa sobre Genoma Humano e Células Tronco do Instituto de Biociências da USP, São Paulo, SP, Brazil. The obtained sequences were assembled with Sequencing Analysis 5.3.1 and submitted for BLAST analysis (Altschul et al. [51]) to infer similarities with *Bartonella* sequences available in GenBank. Different haplotypes were visually discriminated after an alignment using the CLUSTAL W algorithm (Thompson et al. [52]) implemented in Geneious R11 [53]. The molecular analysis for the dipteran was performed in the same way that the mites’ DNA was extracted. Additionally, the material was tested for the same bacteria using the protocols used for ticks and mites, as described above.

### 2.5. Ethical Aspects

This study was approved by the Biodiversity Authorization and Information System (SISBio) of the Instituto Chico Mendes de Conservação da Biodiversidade—ICMBio (No. 77013) and the capture procedures were in accordance with the resolutions of the Animal Experimentation Ethics Committee of ICB/USP (approved under CEUA No. 7946291123).

## 3. Results

After 75 nocturnal samplings, with a total sampling effort of 157,500 m^2^/h, 217 individual bats were captured, belonging to 23 species and six families, as shown in Table 1.

**Figure 2:**
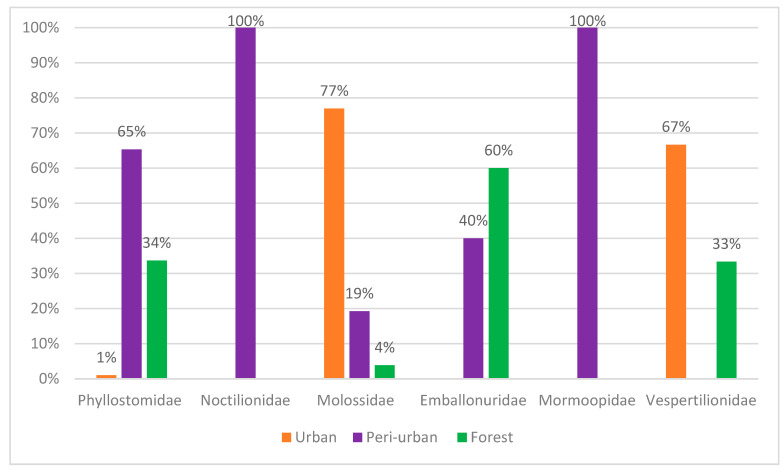
Relative abundance of chiropteran families captured in the sampled areas.

Ten percent of the total number of bats were captured in urban areas (Figure 2). Of these, 4% belonged to the Phyllostomidae family, 9% to the Vespertilionidae family and 87% to the Molossidae family. These bats were captured in residential penthouses through active searches of empty homes and offices.

Sixty seven percent of specimens were captured in peri-urban areas (Figure 2). Of these, 46% belonged to the Phyllostomidae family and 37% to the Noctilionidae family. The families Emballonuridae, Mormoopidae and Molossidae were responsible for 7%, 6% and 4% of captures, respectively.

Twenty-three percent of the specimens were captured in forest fragments (Figure 2). The Phyllostomidae family represented 66% of the captures, followed by the Emballonuridae family with 30%. The Molossidae and Vespertilionidae families represented only 2% each, considering the total sampling efforts.

A total of 592 specimens of ectoparasites were collected from 14 bats and identified into seven families, as shown in Table 1. Overall, 37% of the bats were infested by at least one type of ectoparasite. Regarding the capture area, 67% of the bats were captured in peri-urban areas, 23% in forests and 10% in urban areas.

Among the 217 captured bats, 23 (10.6%) were found to be infested by ticks. A total of 308 ticks were collected, giving a mean intensity of infestation of 13.4 ticks/infested bat. In most of the infested bats, less than 10 ticks were collected; however, in two bats, a total of 87 and 100 ticks were collected. The 308 ticks were morphologically identified as *Ornithodoros hasei* (302 larvae collected from 20 *Noctilio leporinus* and one *Pteronotus rubiginosus*) and *Ornithodoros marinkellei* (six larvae collected from three *P. rubiginosus*). One *P. rubiginosus* was co-infested by the two tick species. Overall, *O. hasei* was collected from 20 (37.7%) out of 53 *N. leporinus* and one (11.1%) out of nine *P. rubiginosus*, whereas *O. marinkellei* was collected from 33.3% (3/9) of *P. rubiginosus.* No ticks were found in the remaining 21 bat specimens that were captured. The most infested bats were *N. leporinus*, with 52% of captures in peri-urban environments, followed by *Peropteryx kappleri*, with 9% also in peri-urban areas, and *Eumops perotis*, with 4% of specimens captured in urban environments. These differences suggest that infestation levels may be related to the type of environment, with a higher infestation observed in bats from peri-urban areas.

Molecular identification of the ticks was confirmed via molecular analyses in 23 *O. hasei* larvae, which generated a single 16S rRNA haplotype (426 bp). Through the BLAST analysis, this haplotype was 100% identical to a sequence of *O. hasei* from southeastern Brazil (KX099896). Two specimens of *O. marinkellei* generated a 16S rRNA haplotype (426 bp) that was 100% identical to a sequence of *O. marinkellei* from Porto Velho, Rondônia (HM582438).

A total of 52 tick samples containing 87 larvae (84 *O. hasei*, 3 *O. marinkellei*) were tested for the presence of DNA of organisms of the genera *Rickettsia*, *Borrelia* and *Coxiella.* Only one larva of *O. hasei* yielded amplicons for the genus *Rickettsia*, via both the real-time PCR and the conventional PCR assays. For the latter assay, a 350-bp fragment of the *gltA* gene was generated, which was 100% identical to the available GenBank sequences of ‘Candidatus *Rickettsia wissemanii*’ from *O. hasei* from French Guiana (MH614266) and the state of Amapá, Brazil (MH614266).

In this study, 17 species of captured bats were infested with parasitic flies (Table 1). A total of 48 bat fly samples were tested for the presence of DNA of *Rickettsia*, *Bartonella*, *Borrelia* and *Coxiella*. All samples were positive for the endogenous control (18S rRNA), validating the DNA extraction protocol. When tested for detection of the DNA of pathogens, two of the 48 bat fly samples were positive for the *nuoG* gene of *Bartonella* spp., while all the samples were negative for the other tested bacterial genera. One sequence for the *nuoG* gene was detected in *T. joblingi* collected on *C. brevicauda*, and another in *S. mirabilis* collected on *T. cirrhosis*. When compared with the sequences available in GenBank, these sequences were 93.44 and 90.57%, respectively, (e-value: 3 × 10^−149^, 1 × 10^−132^; query cover: 100%) and identical to *Bartonella* spp. from *D. ecaudata* blood collected in São Paulo, Brazil [50] (GenBank accession numbers PP445025 and PP445026).

In addition, 27 individual bat-associated mites (4 Trombiculidae, 1 Macronyssidae and 22 Spinturnicidae) were tested. All the samples were positive for the endogenous control (18S rRNA). The results show differences in dipteran infestation between peri-urban and forest environments. In the peri-urban area, *Carollia brevicauda* presented a significantly higher infestation (77%) by *Trichobius joblingi* and *Strebla guajiro*. In the forest area, *Trachops cirrhosus* had a lower infestation (11%) by *Strebla mirabilis*. In general, dipteran species associated with bats were similar between forest and peri-urban environments.

## 4. Discussion

This study resulted in the capture of 217 bats, distributed across 23 species and six families. Most captures occurred in peri-urban areas (67%), followed by forest fragments (23%) and urban areas (10%). The Molossidae family was predominant in urban areas, representing 87% of captures, while in peri-urban areas, Phyllostomidae was the most representative with 46%, followed by Noctilionidae with 37%. In forest fragments, Phyllostomidae was also the most common, with 66% of captures.

We provide the first report of *O. hasei* on *P. rubiginosus*, which has been reported as the main host for *O. marinkellei* [37]. We detected the presence of ‘Candidatus *R. wissemanii*’ in only one specimen of *O. hasei*, giving a minimum infection rate of 1.2% (1/84). Previous studies reported this rickettsial agent in one out of three pools of *O. hasei* larvae from the state of Amapá, eastern Brazilian Amazon [54], in 28.9% (31/107) of *O. hasei* larvae from French Guiana [55], and in three *O. hasei* larvae from Argentina [56]. Our report is the first for the western Amazon. Although ‘Candidatus *R. wissemanii*’ is a member of the spotted fever group of the *Rickettsia* species, its pathogenic role in humans or animals remains to be evaluated [37,54,55,56,57,58]. Finally, Tahir et al. [55] in French Guyana, also tested t *O. hasei* ticks for *Borrelia* and *Coxiella* DNA; similarly to the present study, no ticks were infected by these agents.

Some *Rickettsia* species are the etiological agents of spotted fever in humans, who acquire the infection through the bite of infected ticks; in Brazil, this is chiefly through ticks of the genus *Amblyomma*. As ‘Candidatus *R. wissemanii*’ has never been associated with disease in humans or animals, it is a novel tick-borne agent that was only recently described [54,55,56]. Therefore, this result should be better investigated in further studies.

*Coxiella burnetii* and *Borrelia* spp. were not detected in this study. There are a few cases of the disease identified in Brazil [59,60,61]. Muñoz-Leal et al. [59], Oliveira et al. [62], and Pacheco et al. [63] Further studies are warranted to verify the circulation of relapsing fever in the state of Rondônia, where the argasid fauna is the richest in Brazil. This indicates the need for more intensive epidemiological surveillance by the governments of Brazil. Serological studies should also be carried out in humans and animals to estimate the burden of the disease.

The role of ticks in the transmission of *Bartonella* spp. is controversial, even though they have been found infected in nature (which does not necessarily class it as a vector). Other arthropods (lice and sandflies) are confirmed vectors. A cat’s scratch and/or bite can transmit the bacteria, as can the saliva, urine, and feces of bats [64]. The occurrence of *Bartonella* spp. has been found in five families of chiropterans [62] and in bat flies of the families Nycteribiidae and Streblidae. Regarding bats from the family Phyllostomidae, Ferreira [65] provided evidence on *Bartonella*, which is also prevalent in populations of bats and their ectoparasites in Brazil, helping to clarify the distribution of *Bartonella* spp. related to bat ectoparasites in South America [66]. In this study, infection by *Bartonella* is recorded for the first time in bat flies (*Trichobius joblingi* and *Strebla mirabilis*) in the Brazilian Amazon. Previously in the Amazon, Morse et al. [67] reported *Bartonella* in parasite flies in French Guyana. In Brazil, the occurrence of infections by *Bartonella* in parasitic bat flies was reported by Braga et al. [66] in the state of Maranhão, and by Amaral [68] in the state of Rio de Janeiro.

Hayman [30] and Subudhi et al. [31] highlight that, in recent years, interest in bat research has increased due to the occurrence of the spillover phenomenon, in other words, the transmission of a pathogen from its natural reservoir or host species to a new host species, thus enhancing the possibility of spreading diseases to humans and other mammals. In Brazil, the number of complaints of human infestations by bat ticks inside urban and rural households has increased substantially in recent years [69,70,71]. Indeed, health authorities should be aware of the possibility of emerging vector-borne diseases linked to bats in Brazil.

Bats of the family Phyllostomidae showed a great richness of species, being captured in the three different areas, with a greater abundance of species observed in peri-urban areas. In total, 13 species were captured. Considering the most-used capture method in this study (mist nets installed at 1.5 m above ground level), the high richness of phyllostomids was predictable, given the greater diversity of these bats in the tropics, as well as their foraging characteristics [35]. However, it is crucial to point out that using only this method may underestimate the number of other families, such as Vespertilionidae and Molossidae [72], and can thus be considered a selective method. Therefore, adopting a combination of methods, such as mist nets (canopy), active searches and harp traps, is recommended to ensure a comprehensive and representative sampling of bats [73,74,75].

Members of the family Molossidae, were identified in 52% (20) of the sampling effort carried out in urban areas, establishing direct contact with human beings when they lodge in the roof spaces of homes. Harboring a diverse microbiota, made up of pathogenic and non-pathogenic agents, the intensification of contact between these bats and humans can result in highly pathogenic zoonoses, however the tests carried out by PCR assays resulted in negative results for pathogens. Factors such as the reduction in food supply and the loss of natural habitat for these bats may lead them to move even closer to human settlements, thus contributing to a higher risk of vector-borne bacteria transmission to humans [31,76].

Two tick species, *O. hasei* and *O. marinkellei*, were found infesting bats in the present study. Previous reports of *O. hasei* in Brazil were in the eastern Brazilian Amazon, in the states of Pará [77] and Amapá [54], and in the Pantanal and Caatinga biomes [57,58]. Therefore, our reports are the first for the western Brazilian Amazon. On the other hand, the present report of *O. marinkellei* expands its geographic range in the state of Rondônia, since previous records of this tick species were restricted to the northern part of the state [37].

Despite the richness of 17 bat species, ticks were found on only two species, *N. leporinus* and *P. rubiginosus.* A previous study reported *N. leporinus* as a host for *O. hasei* in the Pantanal biome [58]; thus, this is the first report for the Brazilian Amazon. Interestingly, the relatively high prevalence (37.7%) of *O. hasei* on *N. leporinus* in the present study is similar to a previous study that reported this tick species infesting 40% of the bat *Artibeus planirostris* in the Caatinga [44]. In the Pantanal biomes, where *O. hasei* was the only tick species associated with bats, Muñoz-Leal et al. [58] proposed that *A. planirostris* was the most important host for *O. hasei*, despite several other infested bat species, including *N. lepori nus*, also being found. Our results suggest that *N. leporinus* is the most important host species for the studied area, in the municipality of Monte Negro, western Brazilian Amazon. The results show that bats in fragmented ecosystems may be exposed to an increase in the quantity and variety of ectoparasites due to the reduction in the diversity of natural predators and the fragmentation of shelters, which increases the proximity between hosts and, consequently, the spread of ectoparasites and pathogens, and in forests they may present a more limited infestation of ectoparasites, as observed [78]. Regarding the families Spinturnicidae, Macronyssidae and Trombiculidae, none of the specimens tested were positive for pathogens, which may indicate that, despite being associated with bats, these ectoparasites may not be involved in the transmission of the pathogens tested or that the prevalence of these pathogens in mites is low [79].

## 5. Conclusions

The Amazon is home to an extraordinary diversity of animals, surpassing any other Brazilian biome; however, the state of Rondônia still lacks comprehensive information about the diversity of bats and their ectoparasites. In this study, we observed 13 species of bats from the Phyllostomidae family, which is notable for its ecological versatility and dietary diversity. Furthermore, we report for the first time in Rondônia the occurrence of *O. hasei* and ‘*Candidatus* Rickettsia wissemanii’. Additionally, bacteria of the genus Bartonella with zoonotic potential were detected in the bats *Carollia brevicauda* and *Trachops cirrhosus*, primarily in rural areas and forest fragments.

Although the presence of *Bartonella* in bats captured in peri-urban areas—where 67% of bats were sampled—highlights the potential risks of transmission between animals and humans, it is essential to carefully consider the role of ectoparasites. The infected carrier ectoparasites found in this study are more likely associated with enzootic transmission of the bacteria within bat populations rather than zoonotic transmission to humans, as these ectoparasites exhibit stenoxenous host specificity. These findings underscore the importance of further investigations into the ecological relationships between bats, ectoparasites, and bacteria in order to better understand the risks of pathogen spillover to humans.

## Figures and Tables

**Table 1:** Bacteria identified in ticks, mites and flies from bats in Monte Negro, Rondônia, Brazil.

Bats		Number		Ectoparasites	Number			Detected Bacteria
		M	F		M	F	L	
**Emballonuridae**								
	Emballonurinae							
	*Peropteryx kappleri* (Peters, 1867)	3	3	*Hooperella* spp.	-	-	56	-
	*Rhynchonycteris naso* (Wied-Neuwied, 1820)	7	12	*Basilia* spp.	1	2	-	-
				*Spinturnix* spp.	7	-	-	-
	*Saccopteryx bilineata* (Temminck, 1838)	-	1	-	-	-	-	-
**Molossidae**								
	Molossinae							
	*Molossus Molossus* (Pallas, 1766)	7	12	-	-	-	-	-
	*Eumops perotis* (Schinz, 1821)	2	5	*Steatonyssus* spp.	-	-	25	-
**Mormoopidae**								
	*Pteronotus rubiginosus* (Wagner, 1843)	8	1	*Trichobius* spp.	9	9	-	*-*
				*Paradyschira* spp.	-	1	-	-
				*Ornithodoros marinkellei*	-	-	6	-
				Ornithodoros hasei *	-	-	10	-
				*Cameronieta almaensis*	23	2	-	-
**Noctilionidae**								
	*Noctilio leporinus* (Linnaeus, 1758)	19	34	Ornithodoros hasei *	-	-	308	(GenBank No. MH614266) Candidatus Rickettsia wissemanii *
**Phyllostomidae**								
	Carollinae							
	*Carollia brevicauda* (Schinz, 1821)	13	21	*Trichobius joblingi*	11	12	-	(GenBank No. PP445025) *Bartonella* spp.
				*Strebla guajiro*	6	8	-	
				*Macronyssus* spp.	6	-	-	-
	*Carollia perspicillata* (Linnaeus, 1758)	1	3	-	-	-	-	-
	Desmodontinae							
	*Desmodus rotundus* (Geoffroy, 1810)	2	-	Trombiculidae	-	-	8	-
	*Diphylla ecaudata* (Spix, 1823)	3	1	-	-	-		-
	Glossophaginae							
	*Glossophaga soricina* (Pallas, 1766)	2	-	*Trichobius dugesii*	-	3	-	-
				*Periglischrus caligus*	1		-	-
	Phyllostominae							
	*Lophostoma silvícola* (d’Orbigny, 1836)	-	2	-	-	-	-	-
	*Phylloderma stenops* (Peters, 1865)	1	-	*Periglischrus torrealbai*	17	4	-	-
	*Phyllostomus hastatus* (Pallas, 1767)	2	-	*Periglischrus acutisternus*	19	4	-	-
	*Phyllostomus latifolius* (Thomas, 1901)	1	-	-	-	-	-	-
	*Trachops cirrhosus* (Spix, 1823)	-	1	*Strebla mirabilis*	4	1	-	(GenBank No. PP445026) *Bartonella* spp.
	Stenodermatinae							*-*
	*Artibeus obscurus* (Schinz, 1821)	12	33	*Periglischrus iheringi*	13	11	-	*-*
	*Vampyrodes caraccioli* (Thomas, 1889)	1	-	*P. iheringi*	2	1	-	-
	*Sturnira lilium* (Geoffroy Saint-Hilaire, 1810)	1	-	*Periglischrus* spp.	1	-	-	-
**Vespertilionidae**								
	Myotinae							
	*Myotis riparius* (Handley, 1960)	1	-	*Macronyssus* spp.	1	-	-	*-*
	Vespertilioninae						-	
	*Lasiurus villosissimus* (Geoffroy St.-Hilaire, 1806)	-	1	-	-	-	-	-
	*Lasiurus* spp. (Gray, 1831)	-	1	-	-	-	-	-
**Total**		86	131		121	58	413	-

* New records for Rondônia. M—male, F—female, L—larvae.

## Data Availability

Data are available from the National Institute of Science and Technology (EpiAmo), Brazilian Government.
